# Acute urinary retention in a 23-year-old woman with mild encephalopathy with a reversible splenial lesion: a case report

**DOI:** 10.1186/1752-1947-5-159

**Published:** 2011-04-20

**Authors:** Makiko Kitami, Shin-ichiro Kubo, Shinichiro Nakamura, Shinji Shiozawa, Hideyuki Isobe, Yoshiaki Furukawa

**Affiliations:** 1 Department of Neurology, Juntendo Tokyo Koto Geriatric Medical Center, 3-3-20 Shinsuna, Koto, Tokyo 136-0075, Japan; 2 Department of Urology, Juntendo Tokyo Koto Geriatric Medical Center, 3-3-20 Shinsuna, Koto, Tokyo 136-0075, Japan

## Abstract

**Introduction:**

Patients with clinically mild encephalitis/encephalopathy with a reversible splenial lesion present with relatively mild central nervous system disturbances. Although the exact etiology of the condition remains poorly understood, it is thought to be associated with infective agents. We present a case of a patient with mild encephalitis/encephalopathy with a reversible splenial lesion, who had the unusual feature of acute urinary retention.

**Case presentation:**

A 23-year-old Japanese woman developed mild confusion, gait ataxia, and urinary retention seven days after onset of fever and headache. Magnetic resonance imaging demonstrated T2 prolongation in the splenium of the corpus callosum and bilateral cerebral white matter. These magnetic resonance imaging abnormalities disappeared two weeks later, and all of the symptoms resolved completely within four weeks. Except for the presence of acute urinary retention (due to underactive detrusor without hyper-reflexia), the clinical and radiologic features of our patient were consistent with those of previously reported patients with mild encephalitis/encephalopathy with a reversible splenial lesion. To the best of our knowledge, this is the first report of acute urinary retention recognized in a patient with mild encephalitis/encephalopathy with a reversible splenial lesion.

**Conclusion:**

Our findings suggest that mild encephalitis/encephalopathy with a reversible splenial lesion can be associated with impaired bladder function and indicate that acute urinary retention in this benign disorder should be treated immediately to avoid bladder injury.

## Introduction

Patients with clinically mild encephalitis/encephalopathy with a reversible splenial lesion (MERS) present with relatively mild central nervous system (CNS) disturbances, such as drowsiness, delirium, ataxia, vertigo, and headache, and usually recover completely within one month without any sequelae [1]. The magnetic resonance imaging (MRI) features of MERS include reversible lesions limited to the splenium of the corpus callosum (SCC) or to the SCC and frontal white matter; hyperintense signals on T2-weighted images (T2WI), fluid-attenuated inversion recovery (FLAIR) images and diffusion-weighted images (DWI); with low apparent diffusion coefficient (ADC) values and hypo- or iso-intense signals on T1-weighted imaging (T1WI) sequences with no contrast enhancement [1-3]. Although the exact etiology of MERS remains poorly understood, MERS is thought to be associated with infective agents such as influenza virus, rotavirus, Epstein-Barr virus, hepatitis A virus, Legionella pneumophila and infective endocarditis [1,2,4,5]. Whereas the reported cases with MERS presented with clinical features of encephalitis/encephalopathy, we report what is, to the best of our knowledge, the first case of MERS with acute urinary retention.

## Case presentation

A 23-year-old Japanese woman consulted a local physician for fever and headache of recent onset. She was treated with oral antibiotics, but had no response. Seven days after the onset of fever, she noticed lower abdominal distention and visited the emergency department of our hospital. Transurethral catheterization revealed residual urine, and an indwelling balloon catheter was inserted into the bladder. She was not constipated. On admission, her main symptoms were fatigue and unsteadiness on walking.

The physical examination was unremarkable except for mild fever of 36.8°C. Neurological examination showed mild confusion and unsteady gait when unassisted, but no definite signs of meningeal irritation or mucocutaneous lesions, including in the perineal region. Patellar tendon reflexes, plantar reflexes and abdominal wall reflexes were diminished bilaterally. Blood chemistry and urine analysis showed no abnormalities. Cerebrospinal fluid (CSF) examination gave a normal cell count of 1/mm^3^, a slight increase in protein content (60 mg/dL; normal range 15-45 mg/dL) and a slight decrease in glucose level of 29 mg/dL (30.5% of serum glucose; normal ranges 50-90 mg/dL and 50-70%). Bacterial smears and cultures prepared from CSF were negative. There were no increases in oligoclonal bands, IgG index (0.56) and myelin basic protein in the CSF. The CSF enzyme immunoassay was negative for IgM antibodies against Epstein-Barr viral capsid antigen, herpes simplex virus type-1, varicella zoster virus, mumps virus and measles virus. PCR of the CSF sample was negative for herpes simplex virus type 1 and tuberculosis. Serology tests were negative for anti-double-stranded DNA, anti-phospholipid, anti-SS-A/-B antibody, perinuclear-anti neutrophil cytoplasmic antibody (ANCA) and cytoplasmic ANCA. Serum antibody tests against gangliosides (GM1, GM2, GM3, GD1a, GD1b, GD3, GT1b, GQ1b, GA1, galactocerebroside) were all negative. Electroencephalography and abdominal ultrasonography were unremarkable. An ovarian cyst was detected by pelvic ultrasonography.

**Figure 1:**
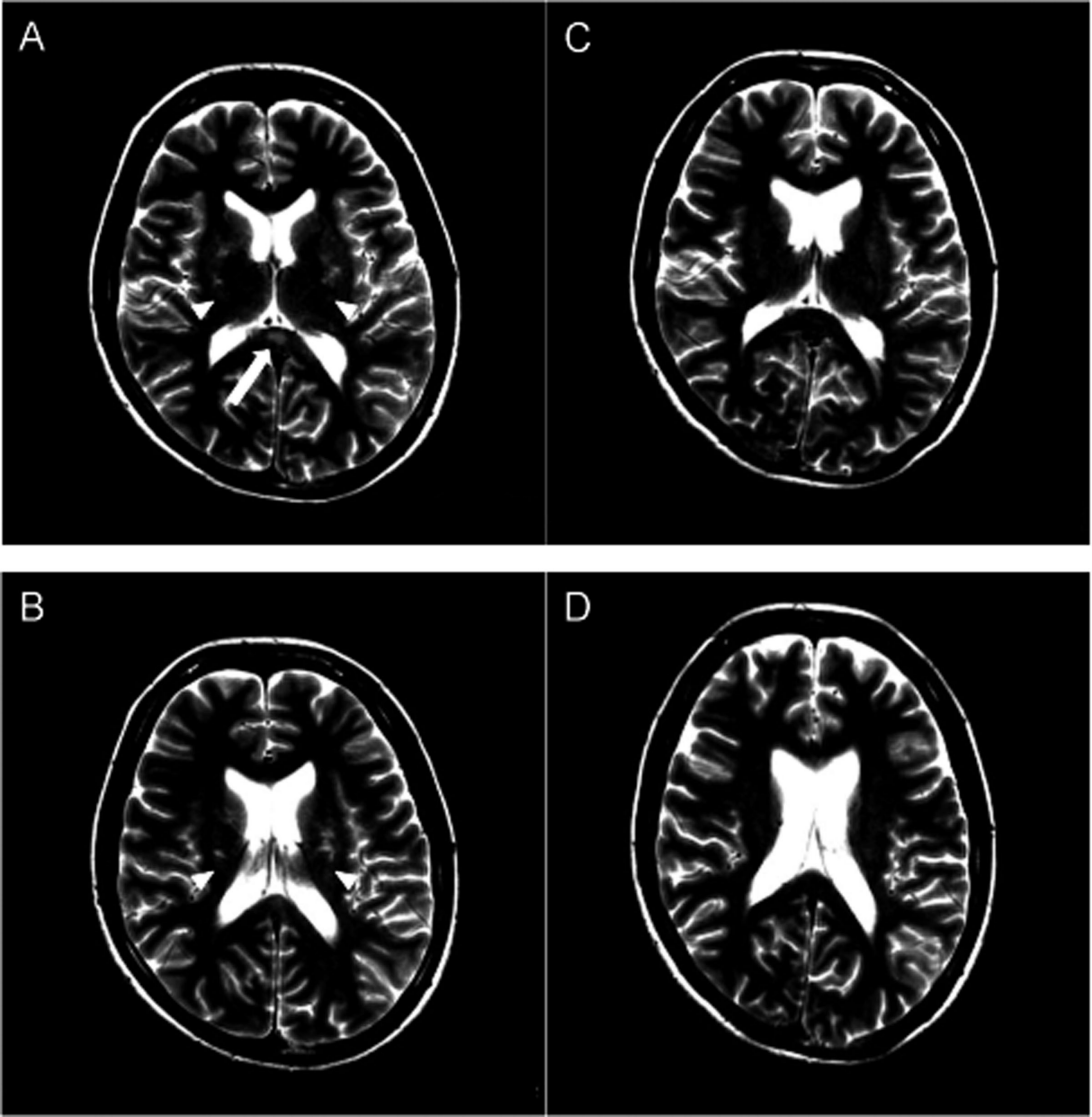
**Mild encephalitis/encephalopathy with a reversible splenial lesion and white matter lesions**. (A,B) T2-weighted images at day five showed a lesion with high signal intensity in the central portion of the splenium of the corpus callosum (SCC) and cerebral white matter lesions (arrow, SCC; arrowhead, cerebral white matter lesions). (C,D) Follow-up MRI scan on day 17 showed no lesions on any sequences (T2-weighted image).

Cranial MRI scans taken on admission showed abnormal signals in the SCC and bilateral cerebral white matter, which were hyperintense on T2WI and FLAIR imaging, and isointense on T1WI sequences with no contrast enhancement (Figure 1). Spinal cord MRI with no enhancement showed no abnormalities. Cystometry showed an underactive detrusor without hyper-reflexia, but bladder sensation was spared, with a first urge to void after intravesical injection of 91 ml of 0.9% saline. Neurophysiological studies revealed nerve conduction of the tibial and median motor nerves and sural sensory nerve that was within the normal range. F-wave was not elicited in the tibial nerves.

We diagnosed our patient with MERS with urinary retention. She was treated with 1 g of methylprednisolone intravenously for three days followed by 60 mg of prednisolone orally for seven days, together with distigmine bromide to treat the urinary retention. A follow-up MRI scan taken 14 days after the initial examination revealed no evidence of the lesions (Figure 1). Our patient's urinary retention and gait ataxia disappeared within four weeks.

## Discussion

In addition to their presence in MERS, reversible focal lesions in the SCC have been reported in patients with seizures, antiepileptic drug toxicity/withdrawal, hypoglycemia, Wernicke encephalopathy, Marchiafava-Bignami disease, sympathomimetic-induced kaleidoscopic visual illusion syndrome, hemolytic uremic syndrome, altitude brain injury and acute axonal injury [4,6]. However, these conditions were ruled out for our patient based on the clinical profile, as there was no history of seizure, hypoglycemia, alcohol and drug use, renal dysfunction, malnutrition or trauma.

MERS was originally reported as a clinicoradiologic finding comprising relatively mild CNS features with complete recovery within one month and MRI findings of a reversible 'isolated' SCC lesion [1]. More recently, symmetric reversible lesions with transient restricted diffusion were reported in the frontal white matter (in addition to the SCC) in some patients with encephalitis/encephalopathy [2,3]. Because the signal characteristics, the reversibility of the frontal and the SCC lesions, and the clinical features of these newly reported patients were identical to those seen in the 'original' MERS presentations, the radiologic spectrum of MERS has been expanded [2,3]. It is therefore reasonable to conclude that the final diagnosis in our patient should be MERS. The cerebral white matter lesions in our patient extended from the corona radiata to the internal capsule, and were different in appearance from the frontal white-matter lesions in the previously reported cases [2,3]. However, the cerebral white-matter lesions in our patient disappeared, together with those in the SCC, without any corresponding features such as pyramidal signs developing, suggesting a pathologic process similar to that of the reported cases, although DWI was not available in our case.

Encephalitis is defined as a brain dysfunction associated with inflammatory changes in the CSF. When there is no evidence of inflammatory changes, the condition can be diagnosed as encephalopathy. We therefore consider that the mild confusion and gait ataxia in our patient were due to encephalopathy. In fact, SCC lesions can present with gait ataxia, as reported previously [4,7,8], although the precise mechanism remains to be clarified.

The acute urinary retention observed in our patient was similar to that described in the meningitis-retention syndrome (MRS) (Table 1), in which urodynamic study revealed a detrusor areflexia. MRS is characterized by aseptic meningitis and acute urinary retention, with no other neurologic abnormalities except for a slightly brisk reflex in the lower extremities [9,10]. MRI of the brain, spinal and lumbar plexus and nerve conduction studies show no abnormalities. CSF examination shows mononuclear pleocytosis, increased protein and normal to mildly decreased glucose content. The clinical course is self-limiting, and complete recovery usually occurs within three weeks. Although the underlying cause remains unknown, parainfectious/autoimmune etiology has been suggested [9,10]. An interesting case was reported recently with clinical features of MRS and reversible SCC lesion [8] (Table 1). That patient exhibited signs and symptoms similar to those of our patient, including fever, headache, gait ataxia and urinary retention. Cystometry also showed a detrusor areflexia. Reversible lesions were detected in the cervicothoracic spinal cord and meninx over the conus medullaris, in addition to the SCC [8].

**Table 1:** Comparison of various disorders characterized by urinary retention.

	Headache, fever	Stiff neck, Kernig sign	DOC	Paralysis	Sensory disturbance	Lower limb reflexes	Ataxia	Urodynamics	Increased cells in CSF	F wave	Brain MRI	Spinal MRI lesion	Revival period
This case	+	-	-	-	Normal	Decreased	+	UD	-	Not elicited	WM, SCC	-	1 month
MERS	+/-	+/-	+/-	-	Normal	Normal or brisk	+/-	-	+/-	Normal	WM, SCC	-	1 month
MRS	+	+/-	-	-	Normal or positive	Normal or brisk	-	UD	+	Normal	-	-	2-10 weeks
Reversible SCC lesion +MRS [8]	+	+	-	-	Normal	Normal	+	UD	+	+	SCC	+	7 weeks
ADEM	+/-	+/-	+/-	Paraparesis or normal	Normal or positive	Normal or brisk or decreased	+/-	DHIC or UD or -	+/-	Normal or not elicited	WM, SCC	+/-	Several weeks or months

ADEM, acute disseminated encephalomyelitis; CSF, cerebrospinal fluid; LL, lower limbs; DHIC, detrusor hyperreflexia with impaired contractile function; DOC, disturbance of consciousness; MERS, mild encephalitis/encephalopathy with a reversible splenial lesion; MRS, meningitis-retention syndrome; SCC, splenium of the corpus callosum; UD, underactive detrusor with or without hyper-reflexia; WM, white matter lesion.

The lesion site responsible for the urinary retention in our patient might have been localized at the conus medullaris to the spinal nerve roots, based on the findings of cystometry, which revealed underactive detrusor without hyper-reflexia. Although data from gadolinium enhancement of the lumbosacral MRI were not available for our patient, the diminished patellar tendon reflex and the absence of F-wave in the tibial nerves might also indicate the involvement of lumbosacral region as the lesions. It seems unlikely that the cerebral white-matter lesions in our patient resulted in her urinary retention because, if these lesions were responsible, the pattern of cystometry would exhibit hyper-reflexia of the detrusor. The fact that our patient is the first reported case with MERS presenting with urinary retention suggests that the SCC lesion may not have been responsible for the urinary retention.

The clinical features of rapid-onset encephalopathy, multiple cerebral lesions and a clinically evident antecedent infection could also suggest acute disseminated encephalomyelitis (ADEM) [11-13]. Patients with ADEM do in fact commonly exhibit urinary dysfunction [14], and acute polyradiculoneuropathy can also occur in ADEM [13]. However, the cerebral lesions in ADEM are typically extensive and asymmetric, although involvement of the corpus callosum is not uncommon [13]. The typical urodynamic findings in ADEM include detrusor hyper-reflexia and impaired contractile function, probably due to suprasacral spinal cord lesion [10]. Further studies are needed to determine whether these parainfectious disorders, including MERS, MRS and ADEM, represent a broad spectrum of the same disease (Table 1). For treatment, corticosteroids are accepted as the drugs of choice for ADEM. By contrast, the effectiveness of steroid treatment is unclear in patients with MRS or MERS, including our patient.

Because our patient was relatively young to have an ovarian cyst, Fowler's syndrome (FS) should be included in the differential diagnosis. FS is a primary disorder of urethral sphincter relaxation in young women, frequently associated with ovarian cysts [15]. However, the urinary retention in FS rarely resolves spontaneously, and usually requires self-catheterization or indwelling suprapubic or transurethral catheters for long periods. No other neurologic deficits have been reported in FS, making it an unlikely diagnosis for our patient.

## Conclusion

MERS can be complicated by acute urinary retention. To avoid bladder injury in this benign condition, symptomatic treatment of the urinary retention with an indwelling catheter is necessary until spontaneous recovery of bladder function occurs.

## Consent

Written informed consent was obtained from the patient for publication of this manuscript and accompanying images. A copy of the written consent is available for review by the Editor-in-Chief of this journal.

## Competing interests

The authors declare that they have no competing interests.

## Authors' contributions

MK, SK, SN, FY analyzed and interpreted the patient data regarding MERS and other diseases. SS and HI performed the urine examination and cystometry, and analyzed the neurogenic bladder. All authors read and approved the final manuscript.
